# Protein language models enable prediction of polyreactivity of monospecific, bispecific, and heavy-chain-only antibodies

**DOI:** 10.1093/abt/tbae012

**Published:** 2024-05-30

**Authors:** Xin Yu, Kostika Vangjeli, Anusha Prakash, Meha Chhaya, Samantha J Stanley, Noah Cohen, Lili Huang

**Affiliations:** Biotherapeutics and Genetic Medicine, AbbVie Bioresearch Center, Worcester, MA 01605, United States; Biotherapeutics and Genetic Medicine, AbbVie Bioresearch Center, Worcester, MA 01605, United States; Biotherapeutics and Genetic Medicine, AbbVie Bioresearch Center, Worcester, MA 01605, United States; Biotherapeutics and Genetic Medicine, AbbVie Bioresearch Center, Worcester, MA 01605, United States; Biotherapeutics and Genetic Medicine, AbbVie Bioresearch Center, Worcester, MA 01605, United States; Biotherapeutics and Genetic Medicine, AbbVie Bioresearch Center, Worcester, MA 01605, United States

**Keywords:** deep learning, protein language model, polyreactivity, polyspecificity, bispecific antibody

## Abstract

**Background:**

Early assessment of antibody off-target binding is essential for mitigating developability risks such as fast clearance, reduced efficacy, toxicity, and immunogenicity. The baculovirus particle (BVP) binding assay has been widely utilized to evaluate polyreactivity of antibodies. As a complementary approach, computational prediction of polyreactivity is desirable for counter-screening antibodies from *in silico* discovery campaigns. However, there is a lack of such models.

**Methods:**

Herein, we present the development of an ensemble of three deep learning models based on two pan-protein foundational protein language models (ESM2 and ProtT5) and an antibody-specific protein language model (PLM) (Antiberty). These models were trained in a transfer learning network to predict the outcomes in the BVP assay and the bovine serum albumin binding assay, which was developed as a complement to the BVP assay. The training was conducted on a large dataset of antibody sequences augmented with experimental conditions, which were collected through a highly efficient application system.

**Results:**

The resulting models demonstrated robust performance on canonical mAbs (monospecific with heavy and light chain), bispecific Abs, and single-domain Fc (VHH-Fc). PLMs outperformed a model built using molecular descriptors calculated from AlphaFold 2 predicted structures. Embeddings from the antibody-specific and foundational PLMs resulted in similar performance.

**Conclusion:**

To our knowledge, this represents the first application of PLMs to predict assay data on bispecifics and VHH-Fcs.

## Teaser

Statement of Significance: Our study yields insights on building infrastructures to support machine learning activities and training models for critical assays in antibody discovery.

## Introduction

There are two types of off-target binding activities for monoclonal antibodies (mAbs): polyspecificity and polyreactivity [1]. Polyspecificity is thought to be driven by factors such as molecular mimicry of the antigens, plasticity of the complementarity-determining region (CDR), and the presence of multiple, possibly overlapping, paratopes. On the other hand, polyreactivity is associated with the presence of excess positive charge or hydrophobicity in the variable region, leading to low-affinity binding with matrix or membrane proteins through general nonspecific chemical interactions. Although polyspecific interactions can sometimes be identified and eliminated, polyreactivity is often of unknown origin, presenting a greater challenge in antibody development [2, 3]. Failure to address polyreactivity could lead to impact on pharmacokinetics (PK), potency, bioavailability, and immunogenicity [1, 2, 4].

Several binding assays have been developed to assess polyreactivity. These assays utilize HEK293 cells, double-stranded DNA (dsDNA), heparin, insulin, bovine serum albumin (BSA), or baculovirus particles (BVPs) [2, 4–7]. In BVP ELISA (or BSA ELISA), the plates were coated with BVP (or BSA). Primary mAbs were then added at high concentrations to the plates and detected using a secondary mAb. Although BSA has widely been used as a nonspecific reagent, BVP ELISA has been shown to correlate with clearance in humans and non-human primates [2], confirming its value for polyreactivity screening. When conducting these polyreactivity assays, high antibody concentration is often necessary due to weak interactions, therefore posing a challenge for small-scale/high-throughput protein production where material is scarce. As such, employing Machine Learning (ML) models to predict polyreactivity and guide the prioritization of clones for scale-up production would be useful. In addition, such models are critical for *in silico* antibody discovery campaigns as models trained on binding data alone lack the ability to predict specificity and therefore a non-specificity model is often required as a counter-screen method [8, 9].

Several models for predicting antibody polyreactivity have been described, utilizing molecular descriptors from sequence and structures [6, 10], embeddings from one-hot residue representation [8, 9], or protein language models (PLMs) [11]. PLMs are models trained on large corpus of sequence data such as the Uniref and Observed Antibody Space. Often built on top of the transformer architecture, they learned to predict masked amino acids from the context of entire sequences, therefore can be used to generate residue-level representations of the biochemical properties, structural features, and evolutionary information about the proteins. Several PLMs, such as Prot5_XL_Uniref [12], ESM2 [13], and Antiberty [14] reached state-of-the-art performance when benchmarked in secondary structure prediction tasks. Outputs from their intermediate layers (a.k.a. hidden layers) can be extracted and potentially used as inputs to train models designed for property prediction tasks other than those in the benchmark studies, in a process called transfer learning or fine-tuning. To our knowledge, no polyreactivity model has been developed using the BVP and BSA assays, nor has embedding from a PLM been used to predict functional properties of unconventional, bispecific formats. Herein, we generated a large BVP and BSA assay dataset augmented with experimental parameters. Using this dataset, we developed an ensemble of three deep learning models based on two PLMs (ESM2 and ProtT5) and one antibody-specific PLM (Antiberty). Our models demonstrated robust performance on polyreactivity prediction for canonical mAbs [monospecific with heavy and light chain (LC)], single-domain Fc (VHH-Fc), and 12 subtypes of bispecific Abs.

## Results

### Exploratory data analysis

#### Description of the July dataset

**Figure 1:**
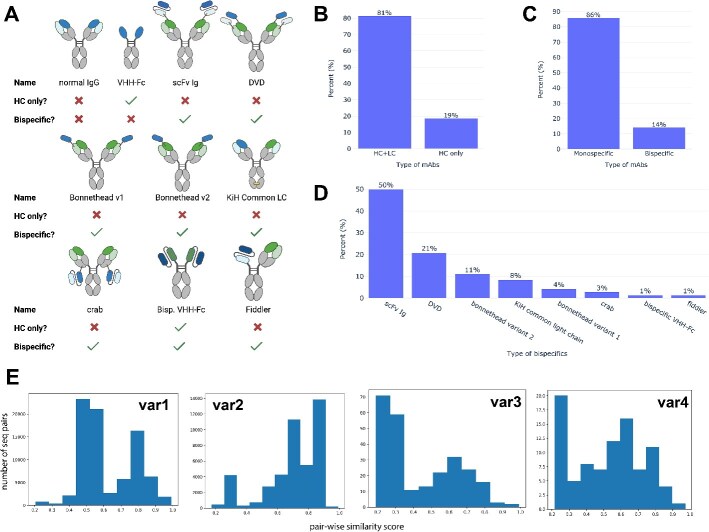
Distribution of *N* = 509 unique mAbs in the July dataset; (A) schematic drawing of mAb types; (B) percentage of HC-only (VHH-Fc) mAbs or mAbs with both HC and LC; (C) percentage of the monospecific (including monospecific canonical mAbs and monospecific VHH-Fc) or bispecific mAbs ; (D) distribution of different bispecific formats, calculated as percentages of the total number of bispecific mAbs; (E) pair-wise sequence similarity scores calculated using biopython package on variable regions var1, var2, var3, and var4; definition of these regions were described in the Method section.

The July dataset consisted of human IgG mAbs mostly in the discovery stage from our internal campaigns against different targets. We defined uniqueness on two levels. The unique mAbs refer to distinct sequences. Among the 509 unique mAbs, 81% had both heavy chains (HCs) and LCs, while the rest were HC only. All HC-only mAbs were VHH-Fc. In addition, 86% were monospecific, with the remaining being bispecific. Of the bispecific mAbs, 50% were in the scFv Ig format, while the others adopted various formats (Fig. 1A–D). The median sequence similarity scores for variable regions 1 through 4 were 0.5 for region var1, 0.7 for region var2, 0.3 for region var3, and 0.6 for region var4, suggesting that the sequences were very diverse, as shown in Fig. 1E.

On the other hand, the unique data points refer to a unique combination of mAb sequence, concentration (ranging from 6.67 nM to 667 nM), and well coating type (either 0.5% BVP or 5% BSA). Unlike most literature models that omit experimental conditions, this approach had three benefits. First, it augmented the dataset from 509 unique mAbs to 1664 unique data points, among which 509 data points were tested against BVP at 667 nM. Second, it reduced noise due to varying experimental conditions. Third, it gave the model an opportunity to learn about concentration-dependent effects of polyreactivity.

#### Concentration-dependent effects of polyreactivity

Polyreactivity assays often require high Ab concentrations, closer to *in vivo* concentrations when administered to humans. We assessed the impact of concentration on BVP and BSA ELISA by comparing the results of 313 unique mAbs tested at both 667 nM (roughly 100 μg/ml for a typical human IgG1) and 66.7 nM. The fold of signal over a negative control Ab was calculated. Based on data from a panel of positive control Abs with varying degree of polyreactivity and known to have clearance and/or toxicity issues, a threshold of 2 was set to distinguish between clean binders and polyreactive binders. Some antibodies showed polyreactivity at both 667 nM and 66.7 nM concentrations (Supplementary Fig. 1A), while some antibodies only appeared polyreactive at the higher concentration (Supplementary Fig. 1B). While not surprising, this result indicates that concentration needs to be taken into consideration when building models, if/when the dataset contains measurements at different concentrations.

#### Correlation of BVP and BSA ELISA

**Figure 2:**
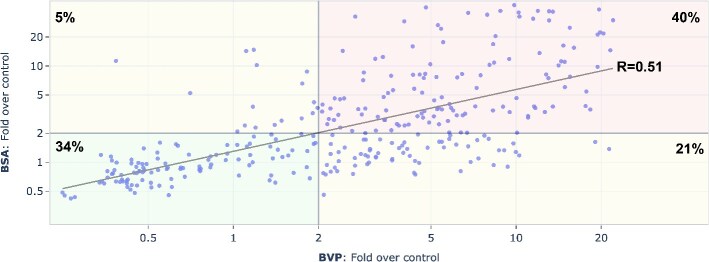
Correlation of BVP and BSA ELISA for 313 unique mAbs at 667 nM. Each dot is a unique mAb; the four quadrants categorize mAbs as clean (fold over control ≤ 2) or polyreactive (fold over control > 2) in one or both assays; percentages of mAbs in each quadrant and the overall Pearson correlation coefficient were indicated on the figure.

A positive correlation was observed between BVP and BSA ELISA for mAbs tested at 667 nM, with a Pearson correlation coefficient (R = 0.51), as shown in Fig. 2. Approximately 74% of mAbs were classified as either clean or polyreactive in both assays (lower left and upper right quadrants in Fig. 2). Approximately 21% of mAbs showed polyreactivity on BVP but not on BSA, while 5% exhibited polyreactivity on BSA but not on BVP. The data indicate that polyreactivity detected by BVP and BSA ELISA has overlapping but different mechanisms.

#### Differences between allele families

We examined the relationship between polyreactivity and allele families of the HC and LC variable regions (a.k.a VH and VL, respectively). One-way Analysis of Variance (ANOVA) analysis on 228 unique monospecific mAbs (canonical mAbs and VHH-Fc) tested at 667 nM in BVP and BSA ELISA showed statistical significance among VH families (*P* < .005). Similar analysis on 134 monospecific mAbs (canonical mAbs only) revealed significance among VL families (*P* < .005) (Supplementary Fig. 2). Although some allele families (e.g. IGHV1–69, IGKV1–27, IGKV1–9) seemed more prone to polyreactivity, the analysis was challenging to interpret. Possible reasons include the lack of consideration for HC and LC pairing, and some allele families having small sample sizes, both of which may confound the analysis. As a result, allele family information alone may not be very useful in predicting polyreactivity.

### Comparison of descriptor model and deep learning model

**Figure 3:**
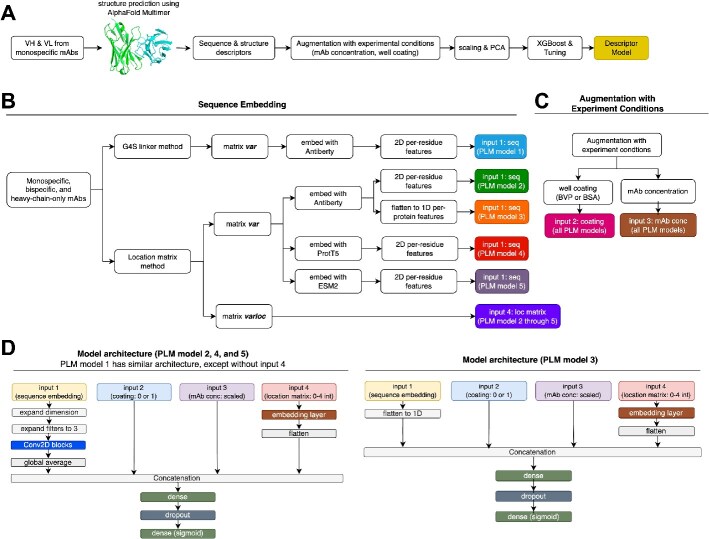
Design of the descriptor model and PLM models; (A) VH/VL pairs from monospecific mAbs were modeled with AlphaFold, sequence, and structure features (a.k.a. descriptors) computed using Schrodinger and merged with experimental condition features; after scaling and PCA, the data were used to train an XGBoost classifier; (B–D) components of the PLM models; (B) blocks labeled “input 1” in distinct colors represent different sequence embeddings generated by Antiberty (antibody-specific PLM), ProtT5 (pan-protein PLM), or ESM2 (pan-protein PLM), as either per-residue or per-protein features; variable domain locations were encoded via either the G4S linker or the location matrix method; (C) “Input 2” and “input 3” represent well coating and mAb concentration, respectively; (D) transfer learning architecture of PLM Model 1, 2, 4, and 5 (left) and 3 (right); the assembled network predicts polyreactivity as either clean (fold over control ≤ 2) or polyreactive (fold over control > 2).

We compared two approaches for polyreactivity prediction: descriptor-based machine learning (a.k.a. the descriptor model) and PLM-based deep learning (a.k.a the PLM models). The descriptor model was built using AlphaFold multimer for VH/VL dimer structure prediction, followed by Schrodinger for sequence and structure descriptor calculation (Fig. 3A). These descriptors compute various sequence- and structure-based features, focusing on individual CDRs, framework regions, or the VH/VL dimer as a whole. Details of these descriptors can be found on Schrodinger’s website. The featurized dataset can be then used as input to build machine learning models using algorithm like XGBoost, support vector machine, random forest, etc. [15]. The PLM Model 1 was trained using embeddings from Antiberty on a transfer learning network composed of 2D convolutional networks and classifier head (Fig. 3B–D), similar to our previous study [16]. Both approaches utilized the July dataset augmented with experimental conditions.

Compared to the PLM Model 1, descriptor model has several drawbacks. First, its application scope is limited to monospecific mAbs due to single VH/VL input constraint. In contrast, PLMs can handle bispecific mAbs by analyzing multiple variable region domains simultaneously. Second, the descriptor model has significantly lower throughput compared to PLMs. Prediction of a single VH/VL pair structure on AlphaFold takes 1–3 h, while pretrained embeddings can be generated within seconds. Finally, when evaluated on blinded data, PLM Model 1 (area under the curve of receiver operating characteristic curve, or ROC AUC: 0.770–0.884) outperformed the descriptor model (ROC AUC: 0.711) in terms of area under the receiver operating characteristic curve (ROC AUC), as summarized in Table 1. One caveat to note that is there are faster structure prediction models such as IgFold [17] that provides similar performance to AlphaFold on Fv modeling, though authors were not able to access at the time of writing due to the need for commercial licenses.

**Table 1:** Summary of model performance in July dataset.

**Model name**	**Params** ^**a**^	**Subset** ^b^	**Data size** ^b^	**% Data split** ^**c**^	**Input feature dimensions** ^**d**^	**Train time (h)** ^**e**^	**Model architecture**				**Performance** **(ROC AUC)** ^f^		
							**Base PLM** ^**g**^	**PLM type** ^h^	**Type of feat.** ^i^	**Location matrix** ^j^	**Train**	**Val**	**Test**
Descriptor model	29 features for XGBoost	Mono-specific	972	80:20	(B, 29)				Per-protein		0.796		0.711
PLM Model 1	6 M	All	1664	80:10:10	(B, 1116, 512, 1), (B, 1), (B, 1)	0.4	Antiberty	antibody-specific	Per-residue	No (G4S was used)	0.903	0.884	0.770
**PLM Model 2**	6 M	**All**	**1664**	**80:10:10**	**(B, 536, 512, 1), (B, 1), (B, 1), (B, 4)**	**0.18**	**Antiberty**	**antibody-specific**	**Per-residue**	**Yes**	**0.908**	**0.871**	**0.826**
PLM Model 3	35 K	All	1664	80:10:10	(B, 536, 512, 1), (B, 1), (B, 1), (B, 4)	0.03	Antiberty	antibody-specific	Per-protein	Yes	0.578	0.612	0.632
**PLM Model 4**	6 M	**All**	**1664**	**80:10:10**	**(B, 532, 1024, 1), (B, 1), (B, 1), (B, 4)**	**0.33**	**ProtT5**	**pan-protein**	**Per-residue**	**Yes**	**0.923**	**0.874**	**0.824**
**PLM Model 5**	6 M	**All**	**1664**	**80:10:10**	**(B, 536, 1280, 1), (B, 1), (B, 1), (B, 4)**	**0.39**	**ESM2**	**pan-protein**	**Per-residue**	**Yes**	**0.946**	**0.906**	**0.838**

a Number of features used in the descriptor model, or the number of trainable parameters in each PLM model.

b Subset denotes whether all data were used, or only a subset of data was used. For the descriptor model, only unique data points from monospecific mAbs were included for training, due to the reasons explained in the text. For the PLM models, all unique data points (canonical monospecific mAbs, bispecific, and VHH-Fc mAbs) were included. Dataset size denotes the total unique data points, with all three splits combined.

c Type of split performed to generate train, validation, and test set, as well as their percentages. The descriptor model has only train and test splits due to its smaller dataset size.

d Shape of input feature matrix. Letter B in the parenthesis denotes the batch dimension. Descriptor model has 1 input of 29 orthogonal eigenvectors after dimensionality reduction by PCA. The PLM models have three to four input matrices as specified.

e The average time to complete 30 epochs was calculated based on three individual experiments.

f Performance on the train, validation, and test dataset measured in ROC AUC. Top three PLM models (PLM Model 2, 4, and 5) were highlighted in bold text.

g Different base PLM models used to generate sequence embeddings.

h The terms *antibody PLM* and *pan-protein PLM* refer to the specificity of the base PLM model, indicating whether it is pretrained on antibodies or general proteins.

i Type of feature column indicates whether 2D convolutional layers were used as per-residues features (e.g. matrices have a dimension representing the number of residues in an antibody, and another dimension representing the features) or 1D layer for per-protein features (e.g. matrices have a dimension of 1 representing the antibody, and another dimension representing the features).

j Whether the location matrix method was used for bispecific mAb embedding. The location matrix method treats each variable domain individually and uses an integer-encoded location matrix to indicate the chain from which each variable region originates. Conversely, the G4S linker method connects multiple variable domains on the same chain using an artificial GGGGS linker, irrespective of the actual connecting regions.

### Optimization of PLM models

#### Embedding bispecific mAbs using G4S linker or location matrix

Embedding bispecific mAbs is challenging because of the different arrangements of the variable domains. As shown in Fig. 1A, in formats like scFv Ig, Dual Variable Domain (DVD), bonnetheads, bispecific VHH-Fc, and the scFv arm of Fiddler, variable domains are connected by short peptides. Conversely, in the crab format, the VL and single-chain variable fragment (scFv) are separated by the LC constant region. Additionally, some formats have bispecific domains only on the HC (e.g. scFv Ig, bonnethead v2, bispecific VHH-Fc), some only on the LC (e.g. bonnethead v1, crab), and some on both chains (e.g. DVD, KiH common LC, Fiddler). A brute-force approach to encode entire HC or LC is impractical, not only due to Antiberty’s 510-amino acid input limitation but also because the large embedding matrices from long sequences would require substantial computational resources for training.

To address this challenge for PLM Model 1, we extracted variable domains on the same HC or LC and then concatenated them using a G4S linker (Fig. 3B). This keeps the input matrix size manageable and retains some positional information. Specifically, the resulting input shapes are (B, 1116, 512, 1) for PLM embedding and (B, 1) each for concentration and coating embeddings, where B is the batch dimension. Although this setup uses the G4S linker to indicate whether two variable domains share the same chain, it lacks detail about which specific chain (e.g. HC1 or HC2) they originate from. In addition, models like Antiberty, ProtT5, and ESM2 are primarily trained on natural protein sequences. Since endogenous mAbs typically feature one variable domain per chain, embedding multiple domains in a single sequence potentially raises questions about suitability.

PLM Model 2 uses an integer-encoded location matrix to identify each variable domain’s origin chain (Fig. 3B). By performing separate embedding on each variable domain, it aligns more closely with training data of Antiberty. In addition, reducing the input feature dimension cut the training time from 0.4 to 0.18 h. Although it slightly lowered validation accuracy from 0.884 to 0.871 (1% decrease), it boosted test AUC from 0.770 to 0.826 (7% increase), prompting us to continue with this approach (Table 1).

#### 2D per-residue feature versus 1D per-protein feature

Both PLM Models 1 and 2 use 2D convolutional neural networks, treating per-residue features as images similar to the approach described in our previous study [16]. An alternative approach, PLM Model 3, flattened these into 1D per-protein features, similar to the descriptor model but without explicit association with sequence and structure features (Fig. 3B). To generate the 1D per-protein features, the embeddings from the PLMs were averaged across the residue dimension. For example, the original embedding matrix in the shape of (batch, residue, feature) was converted to a shape of (batch, 1, feature). PLM Model 3 underperformed across all dataset splits (Table 1), showing significantly worse performance (up to 36% decrease in AUC) compared to PLM Models 1 and 2, as well as the descriptor model. This highlights the importance of per-residue features and convolutional networks in our case.

#### Antibody-specific PLM versus pan-protein foundational PLMs

Switching from antibody-specific PLM Antiberty to pan-protein foundational PLMs like ProtT5 and ESM2 resulted in similar performance. PLM Model 4 and 5 have similar convolutional blocks as PLM Model 2, as shown in Fig. 3D. PLM Model 4, which utilized ProtT5, had an AUC of 0.874 on the validation set and 0.824 on the test set. PLM Model 5, which was built from ESM2, yielded an AUC of 0.906 on the validation set and 0.838 on the test set. This suggests that when used as embeddings, PLMs pretrained on antibody sequences might not necessarily offer a significant advantage over those pretrained on general proteins.

### Performance of PLM Model 5 on subsets of monospecific, bispecific, and VHH-Fc

**Figure 4:**
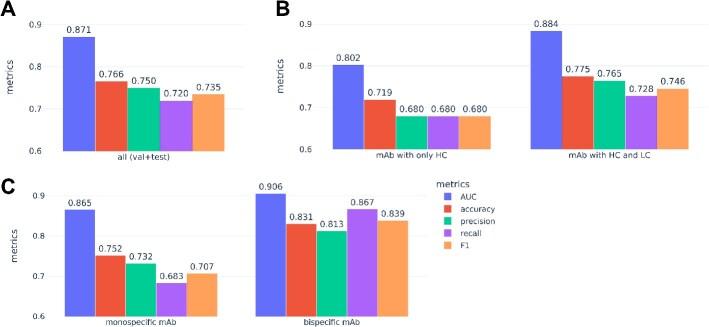
Performance of PLM Model 5 on the combined validation and test sets (*N* = 333 blinded data points) from the July dataset; a panel of metrics was shown in different colors; (A) overall performance; (B) performance on HC-only mAbs (VHH-Fc, *N* = 57) and mAbs with both HC and LC (*N* = 276); (C) performance on monospecific (*N* = 274) and bispecific mAbs (*N* = 59); monospecific mAbs include both canonical IgG as well as monospecific VHH-Fc.

Using the July datasets, we further assessed the performance of PLM Model 5 on the combined validation and test sets, which the model was not trained on. The analysis in Fig. 4A–C provided a breakdown of its efficacy across different types of mAbs. The overall AUC was 0.871, with an AUC of 0.884 for mAbs containing both HC and LC, compared to 0.802 for VHH-Fc. The model performed robustly on bispecific mAbs (AUC 0.906) and monospecific mAbs (AUC 0.865).

### Confirmation of findings with the September dataset

#### Description of the September dataset

**Figure 5:**
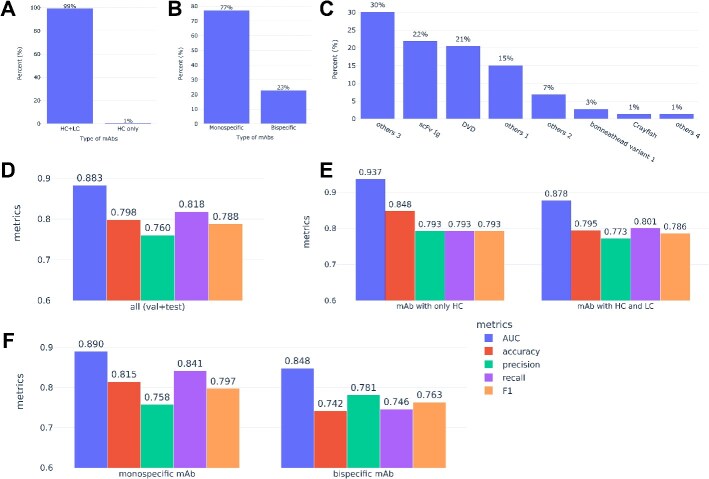
Description of the new data in the September dataset and the performance of the ensemble model; (A–C) distribution of *N* = 321 unique new mAbs added during a 2-month period; (A) percentage of HC-only (VHH-Fc) mAbs or mAbs with both HC and LC; (B) percentage of the monospecific or bispecific mAbs; monospecific mAbs include both canonical IgG and monospecific VHH-Fc; (C) distribution of different bispecific formats; others 1 through 4 refer to undisclosed bispecific formats that did not exist in the July dataset; (D–F) performance of the ensemble model built from PLM 2, 4, and 5 on the combined validation and test sets (*N* = 600 blinded data points) from the September dataset; (D) overall performance; (E) performance on the HC-only mAbs (*N* = 79) and mAbs with both HC and LC (*N* = 521); (F) performance on monospecific (*N* = 480) and bispecific mAbs (*N* = 120).

Our application collected 321 unique mAbs during a period of ~2 months, including 282 that were not presented in the July dataset. This new batch differed notably from the original July dataset. For example, 99% had both HC and LC (Fig. 5A). About 23% were bispecific, including four new undisclosed subtypes (Fig. 5B and C). Due to these differences, the new data were combined with the original July dataset to form the September dataset, totaling 2998 unique data points augmented with experimental conditions. The median sequence similarity score for var1, var2, var3, and var4, as well as the distribution of these scores, was similar to the July dataset.

#### Performance of the PLM models and the ensemble

We fine-tuned PLM Models 2 (Antiberty-based), 4 (ProtT5-based), and 5 (ESM2-based) on the September dataset using a similar approach as before. All models showed robust overall performance, with AUCs ranging from 0.862 to 0.891 on unseen data in the validation and test sets (Table 2). This again confirmed embeddings from antibody-specific PLMs performed comparably to those from foundational PLMs. Each model also maintained strong performance (AUC 0.812–0.968) across different mAb categories: monospecific, bispecific, and VHH-Fc (Supplementary Table 1). Ensemble of PLM Models 2, 4, and 5 further improved some of these metrics, with final AUC values ranging from 0.848 to 0.937 across different mAb formats (Fig. 5D–F).

**Table 2:** Summary of model performance in September dataset.^a^

**Model name**	**Subset**	**Size**	**% Dataset split**	**Train time (h)**	**Base PLM**	**Performance in (ROC AUC)**		
						**Train**	**Val**	**Test**
PLM Model 2	All	2998	80:10:10	0.3	Antiberty	0.950	0.883	0.862
PLM Model 4	All	2998	80:10:10	0.54	ProtT5	0.933	0.891	0.862
PLM Model 5	All	2998	80:10:10	0.66	ESM2	0.901	0.878	0.863

a Column interpretations are consistent with those in Table 1.

The Antiberty-based PLM Model 2 was also further evaluated on a set of 13 approved antibodies on both BVP and BSA ELISA at an antibody concentration of 667 nM, reporting an accuracy score of 0.84 (Supplementary Table 2).

### Mechanistic insights

Prior studies have reported the important role of hydrophobicity [1, 18, 19] and charge [1, 6, 19, 20] in polyreactivity. In BSA ELISA, it is possible that the detected polyreactivity of mAbs is driven by charge, since BSA has an isoelectric point (pI) of around 5 and is therefore negatively charged in pH 7.4 buffer. Data from BSA assay correlate well with that from other negatively charged ELISA such as dsDNA assay (data not shown). The GP64 trimer envelope protein of BVP [21, 22], modeled via AlphaFold, displayed hydrophobic regions along its length. The tip of the trimer presented an area of strong hydrophobic surface (Supplementary Fig. 3). These regions could potentially be associated with hydrophobicity-driven polyreactivity. In addition, wells in BVP ELISA were coated with BVP but blocked with BSA. It is therefore conceivable that the detected polyreactivity in BVP ELISA could be driven by hydrophobicity and/or charge. These observations are corroborated by the SHapley Additive exPlanations (SHAP) scores from the descriptor model. According to the SHAP scores, the top contributing features were mAb concentration, hydrophobicity (LC CDR1), well coating, and positive charges (HC CDR1 and LC FR3, Supplementary Fig. 3). Notably, the highest SHAP score (0.519) was attributed to mAb concentration, confirming the importance of this factor in predictive modeling.

## Discussion

There are several merits of our study. First, it pioneers the application of PLM embeddings to diverse mAb formats like monospecific, bispecific (12 subtypes), and VHH-Fc. By demonstrating uniform embeddings and simultaneous training across these mAb types, we took a step to address the growing interest in developing such formats as therapeutics or tool reagents. Second, we directly compared descriptor model and PLM models, illustrating their complementary nature. The PLM models had a broader application scope and achieved better performance. On the other hand, the descriptor model yielded mechanistic insights that is consistent with empirical observations (e.g. concentration dependent effect) and literature data (e.g. hydrophobicity and charge as key factors driven polyreactivity).

There are several caveats of our models. First, our current model handles up to four variable regions in a single mAb. Second, in either the G4S linker method or the location matrix method, the connection region between the variable domains is lost. Because these regions could be artificial linkers of different lengths, or constant regions of the heavy or LC, they contain important spatial and steric information. Third, the Fc region was not included in the embedding due to its general similarity and the input length restriction from the PLMs. The impact of mutations in the human IgG1 Fc region on BVP/BSA ELISA remains unclear. Positive charges in the variable domain, which could drive polyreactivity detectable by BVP/BSA ELISA, have been shown to directly affect FcRn-dependent PK [1, 6]. Interplay of these factors (charge, hydrophobicity, and Fc mutations) on the polyreactivity and PK requires further investigation. Fourth, currently the PLMs do not encode posttranslational modification (PTM) features. PTMs like sulfation and glycosation can alter antibody structure and paratopes, therefore potentially introducing changes to polyreactivity. Finally, the feature matrix is still far from complete. Factors absent from experiment metadata such as production cell line, protein purity, buffer composition, and freeze–thaw cycle could affect assay readout. In additions, assays that are related to hydrophobicity (e.g. retention time in hydrophobic interaction chromatography a.k.a HIC) and stability (e.g. affinity-capture self-interaction nanoparticle spectroscopy a.k.a. AC-SINS, dynamic light scattering a.k.a. DLS) might be useful input features for polyreactivity models.

In conclusion, we developed an ensemble of models based on antibody-specific and foundational PLM embeddings to predict the polyreactivity in mAbs. These models can be potentially used as counter selection in combination with affinity models for in silico screening of antibodies with desired properties. Our study yields valuable insights on building infrastructures to support machine learning activities and training deep learning models for critical assays in antibody discovery.

## Materials and methods

BVP ELISA was adapted from Hotzel *et al*. [2]. BSA ELISA was developed as a complementary assay to BVP assay and implemented conveniently in the same high throughput experiment. The assays were extensively optimized for automation on EP Motion liquid handler (Eppendorf). Briefly, BVP particles (LakePharma, Cat# SR-17000) were coated on half of a 384-well plate (Greiner Bio, Cat# 781061) at a 0.5% concentration in 50 mM sodium bicarbonate buffer (Pierce, Cat# 28382). The other half of the plate was coated with just the sodium bicarbonate buffer. After overnight incubation at 4 °C, plates were washed with 1× TPBS (1× PBS with 0.05% Tween 20) and blocked with 5% BSA in 1× PBS for 1 h at room temperature (RT). mAbs were added at 25 μl/well in duplicates to the BVP and BSA-coated wells. Although the standard mAb concentrations were 667 nM and 66.7 nM, when materials were scarce, adjustments were made to these concentrations, resulting in a range from 6.67 nM to 667 nM in our datasets. Plates were incubated for an hour at RT, washed three times, and secondary antibody (Thermo, Cat# 31412) was added at a 1:10 000 dilution in 1% BSA in 1× PBS. After 45 min of incubation at RT, plates were washed, and TMB substrate (Thermo, Cat# 34029) was added at 25 μl/well and incubated for 3 min at RT. Stop solution (VWR, Cat# BDH7500–1) was added at 25 μl/well. Readings were taken on a Clariostar Plus microplate reader (BMG Labtech). A low-polyreactive human IgG1 served as the normalization control mAb in each experiment. Given a specific mAb concentration and type of coating, fold over control was calculated as the ratio of the average signal of the sample mAb to that of the control mAb.

### Acquisition of the July dataset and exploratory data analysis

The July dataset was collected using an internal Streamlit application developed by the authors. The data came from BVP and BSA assays conducted primarily for mAbs in the discovery stage. Using Python packages abnumber (v0.3.2) and anarci (v2020.04.23), VH and VL domains were extracted from IgG HC and LC to calculate the number of variable domains, chain stoichiometry, and germline families. These data were used to classify mAbs as monospecific, bispecific, or heavy-chain-only (VHH-Fc). Subtypes of bispecific mAbs (e.g. scFv-Ig, DVD, etc.) were manually annotated. Statistical analysis was generated using scipy (v1.11.1), and figures generated using plotly (v5.16.1) and matplotlib (v3.7.1). Notably, a “unique mAb” is defined by its unique sequence and was utilized in analysis such as mAb type distribution, BVP and BSA ELISA correlation, and germline family data correlation. Conversely, “unique data point” is defined by a unique mAb sequence combined with mAb concentration and well coating. This was used for analyzing concentration effects and training descriptor and PLM models with experimentally augmented datasets.

### Training the descriptor model on the July dataset

A total of 972 unique data points on monospecific mAbs from the July dataset were utilized for building the descriptor model. These mAbs were modeled as VH/VL heterodimers using AlphaFold 2 (v2.3.0), with the top-ranked relaxed structures selected for 910-descriptor computation in Schrodinger Maestro (v2022-3). The resulting feature matrix was combined with experimental data (mAb concentration and coating), standardized, and underwent principal component analysis, yielding 29 orthogonal vectors for XGBoost input. Parameter tuning involved 500 trials on an 80/20 train-test split, and performance was assessed via fivefold cross-validated (5CV) ROC AUC scores. A diagram of the process is shown in Fig. 3A.

### Training the PLM models on the July dataset

A total of 1664 unique data points on monospecific, bispecific, and heavy-chain-only mAbs from the July dataset were utilized for building the PLM Model 1 through 5. The sequence preprocessing involves three steps: stoichiometry-aware chain preprocess, variable region extraction, and variable region embedding. First, for stoichiometry-aware chain preprocessing, we denote HC 1, LC 1, HC 2, LC 2 sequences of a mAb as HC1, LC1, HC2, and LC2. For example, a canonical IgG is processed into a unique non-null HC1, a unique non-null LC1, a null HC2 and a null LC2, as shown in Supplementary Fig. 4A. As another example, a KiH is processed as a unique non-null HC1, a unique non-null LC1, a unique non-null HC2, and a null LC2. In the second preprocessing step, abnumber (v0.3.2) and anarci (v2020.04.23) were used to extract the variable domains from the HC1, LC1, HC2, and LC2. In the G4S linker method, a G4S linker was added to the variable regions from the same chain, as shown in Supplementary Fig. 4B. In the location matrix method, no linker was added. Instead, a location matrix with values from 0 to 4 was generated to denote where the variable regions (var1, var2, var3, var4) came from, as shown in Supplementary Fig. 4C. Any sequence from the July and September datasets was guaranteed to have a non-null var1 and at most four non-null var_i_. In the final preprocessing step, PLM base models Antiberty [14], Prot5_XL_Uniref [12], ESM2 [13] were used to generate per-residue embeddings, as per the default guidelines in their github repositories. The concentration matrix was normalized to a number between 0 and 1 using the following formula:


(1) \begin{equation*} NormalizedConc=1-\frac{\log nM}{\log 667} \end{equation*}

Where $nM$is the original concentration in nM. The coating matrix was encoded 0 if 5% BSA was coated on wells and 1 if 0.5% BVP was coated. The label matrix was encoded 0 if signal over control mAb was less than or equal to 2 and 1 otherwise. These matrices were fed into a deep learning model. An 80/10/10 random split was used to generate train, validation, and test sets. The model was trained only on the train set, using an Adam optimizer with custom learning rate scheduler consisting of 10 epochs of linear warmup, 10 epochs of constant rate at 1e-3, and 10 epochs of cosine decay. The ROC AUC, accuracy precision, recall, and F1 metrics were calculated using sklearn package (v1.3.0) and plotted using Plotly (v5.14.1). A sketch of this process is shown in Fig. 3B–E. Example of training script using Antiberty embedding is provided in Github https://github.com/xinyu-dev/bvp_manuscript.

### Building the ensemble model on the September dataset

The September dataset was formed by combining the July dataset with new entries collected over a 2-month period. We applied an 80/10/10 train-validation-test split, blinding the validation and test sets during tuning. Individual PLM Models 2, 4, and 5 were first individually tuned, then combined into an ensemble using soft voting. The highest-AUC model received a 0.5 weight and others 0.25. Details of training and evaluation process were consistent with prior methods.

### Mechanistic studies

Feature importance from the descriptor model was calculated using the SHAP scores [23]. GP64 trimer envelope protein of BVP was modeled using AlphaFold multimer.

## Supplementary Material

Supp_Figure_1_tbae012

Supp_Figure_2_tbae012

Supp_Figure_3_tbae012

Supp_Figure_4_tbae012

Supp_Table_and_Figure_Legend_tbae012

Supp_Table_2_tbae012

## Data Availability

The datasets were not released due to confidential information. Example training scripts for the PLM models were provided in https://github.com/xinyu-dev/bvp_manuscript.
