# Supplementary figures and images for: Protein language models enable prediction of polyreactivity of monospecific, bispecific, and heavy-chain-only antibodies
Source: Antib Ther. 2024 May 30;7(3):199–208. doi: 10.1093/abt/tbae012 (PMC11259759; doi:10.1093/abt/tbae012)

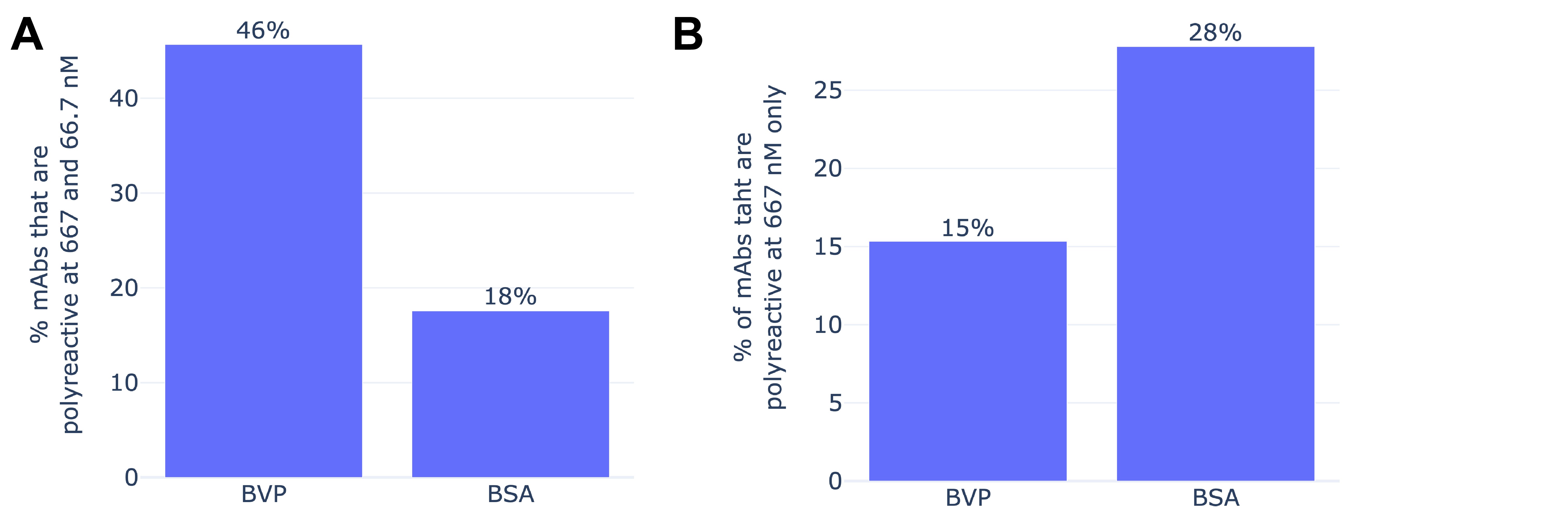

Supplement: Supp_Figure_1_tbae012 [file supp_figure_1_tbae012.jpeg]

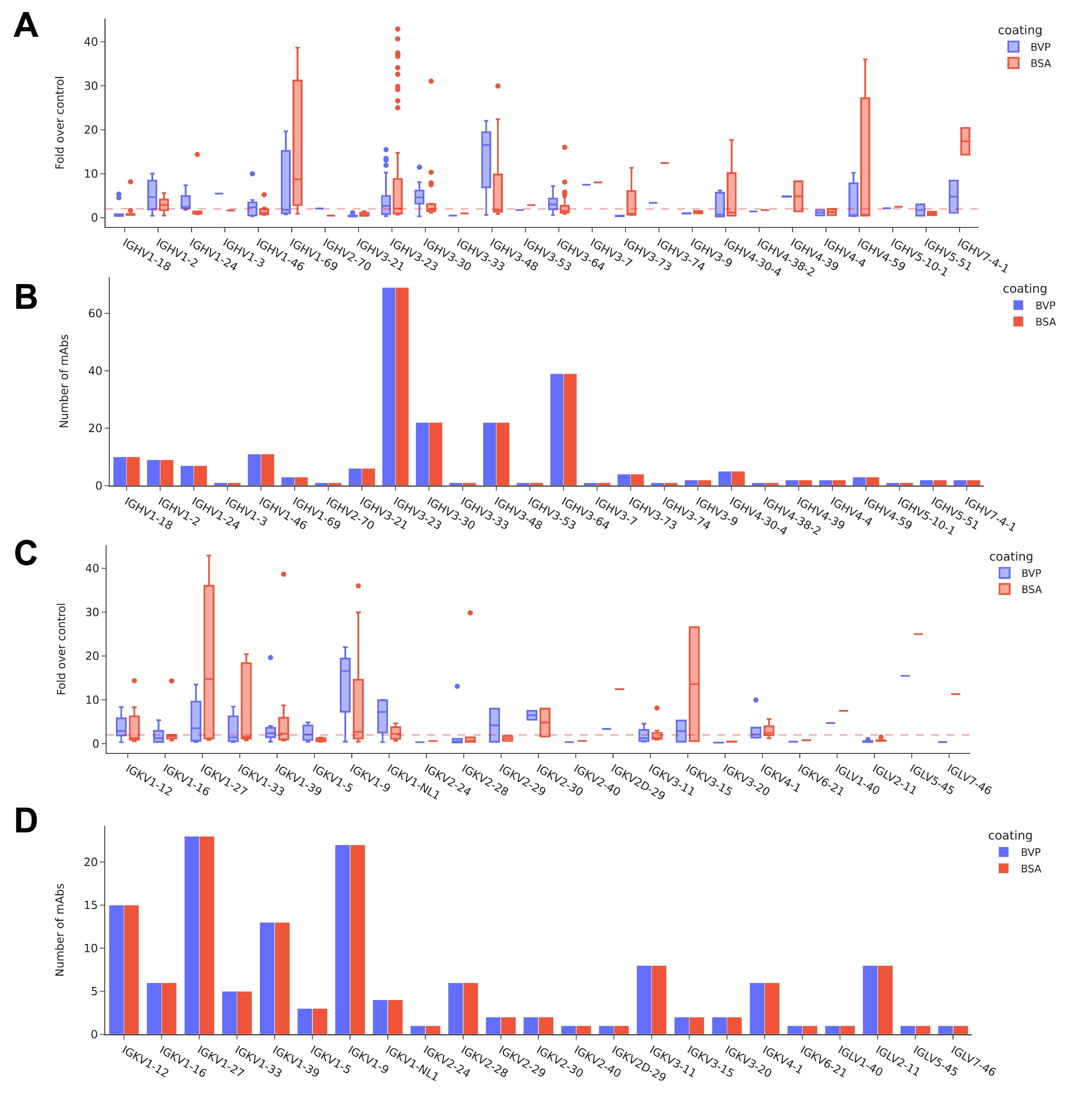

Supplement: Supp_Figure_2_tbae012 [file supp_figure_2_tbae012.jpeg]

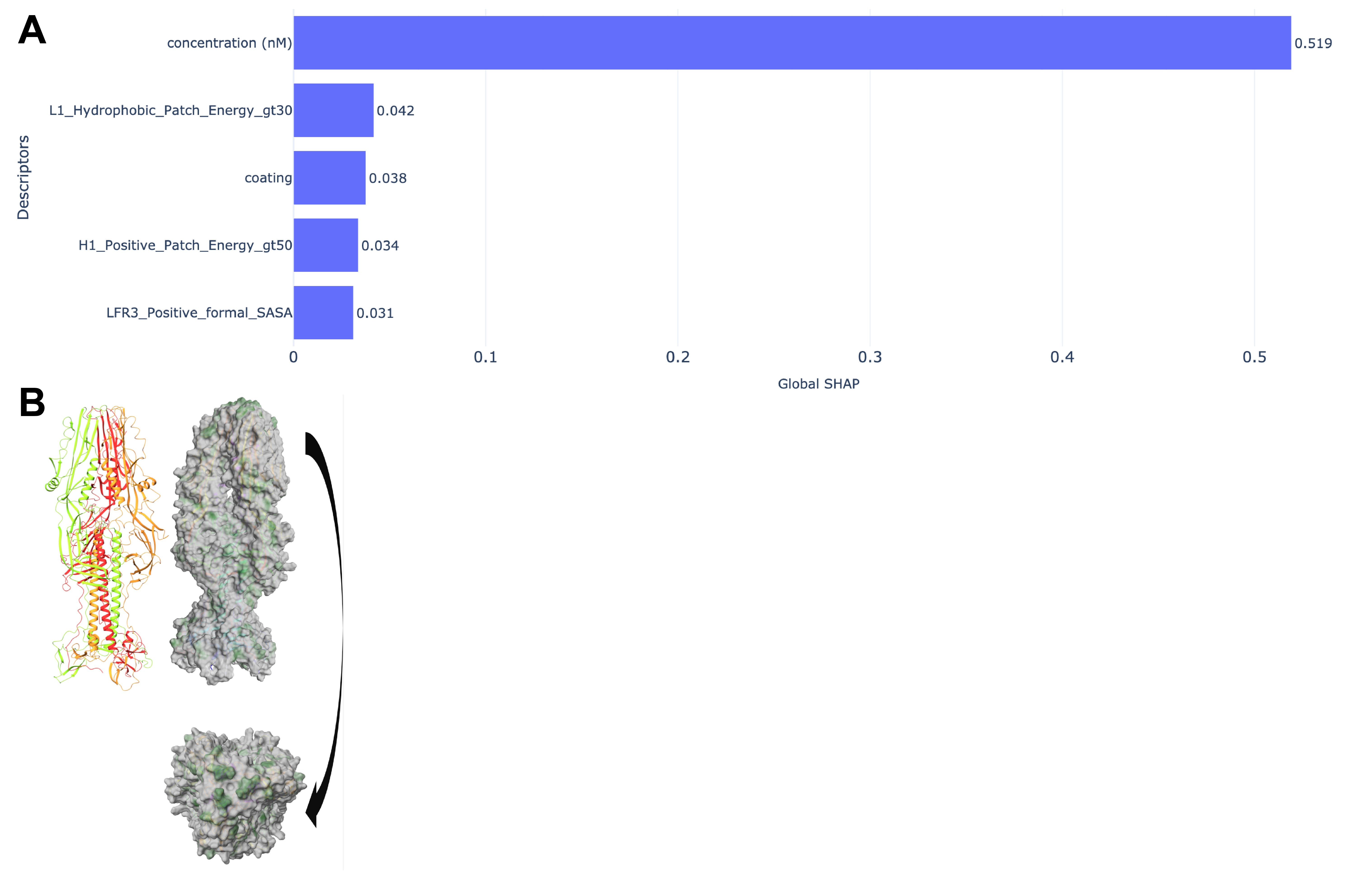

Supplement: Supp_Figure_3_tbae012 [file supp_figure_3_tbae012.jpeg]

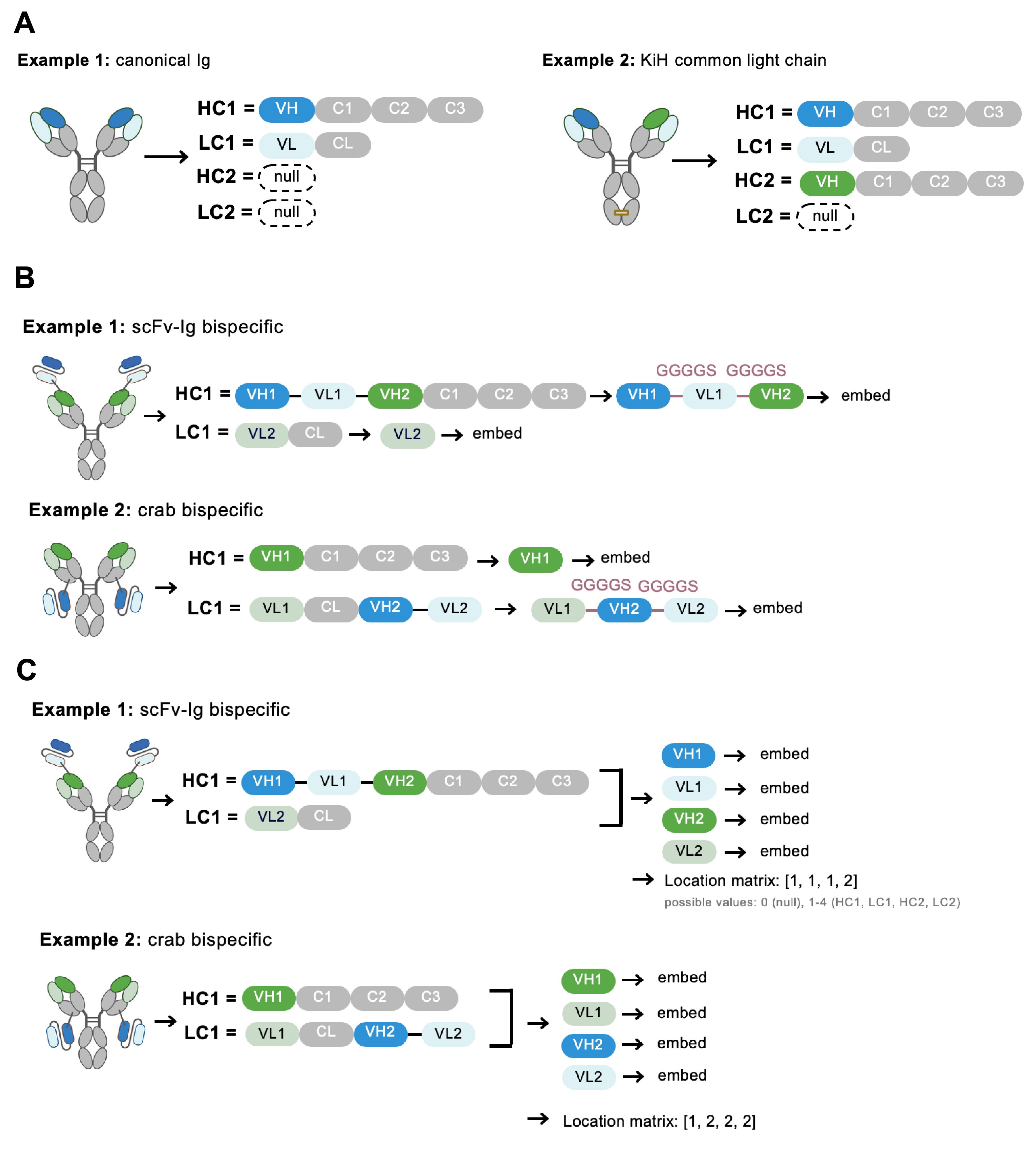

Supplement: Supp_Figure_4_tbae012 [file supp_figure_4_tbae012.jpeg]
