# Supplementary material for: Protein language models enable prediction of polyreactivity of monospecific, bispecific, and heavy-chain-only antibodies
Source: Antib Ther. 2024 May 30;7(3):199–208. doi: 10.1093/abt/tbae012 (PMC11259759; doi:10.1093/abt/tbae012)
Supplement: Supp_Table_and_Figure_Legend_tbae012 [file supp_table_and_figure_legend_tbae012.docx]

Supp. Table 1. Performance of PLM models and ensemble on subsets of the September dataset

| **Model** | **Subset^1^** | **Size** | **ROC AUC^2^** | **Accuracy** | **Precision** | **Recall** | **F1** |
| --- | --- | --- | --- | --- | --- | --- | --- |
| PLM Model 2 | mAb with only HC | 79 | 0.841 | 0.797 | 0.741 | 0.690 | 0.714 |
|  | mAb with HC and LC | 521 | 0.876 | 0.787 | 0.773 | 0.776 | 0.775 |
|  | monospecific mAb | 480 | 0.873 | 0.802 | 0.765 | 0.784 | 0.774 |
|  | bispecific mAb | 120 | **0.862** | 0.733 | 0.787 | 0.716 | 0.750 |
|  | all (val+test) | 600 | 0.873 | 0.788 | 0.770 | 0.767 | 0.769 |
| PLM Model 4 | mAb with only HC | 79 | **0.968** | 0.886 | 0.778 | 0.966 | 0.862 |
|  | mAb with HC and LC | 521 | 0.864 | 0.775 | 0.728 | 0.837 | 0.779 |
|  | monospecific mAb | 480 | 0.889 | 0.808 | 0.730 | 0.885 | 0.800 |
|  | bispecific mAb | 120 | 0.812 | 0.717 | 0.746 | 0.746 | 0.746 |
|  | all (val+test) | 600 | 0.877 | 0.790 | 0.734 | 0.851 | 0.788 |
| PLM Model 5 | mAb with only HC | 79 | 0.916 | 0.785 | 0.800 | 0.552 | 0.653 |
|  | mAb with HC and LC | 521 | 0.865 | 0.781 | 0.789 | 0.732 | 0.759 |
|  | monospecific mAb | 480 | 0.879 | 0.802 | 0.809 | 0.712 | 0.757 |
|  | bispecific mAb | 120 | 0.808 | 0.700 | 0.738 | 0.716 | 0.727 |
|  | all (val+test) | 600 | 0.870 | 0.782 | 0.790 | 0.713 | 0.750 |
| Ensemble | mAb with only HC | 79 | 0.937 | 0.848 | 0.793 | 0.793 | 0.793 |
|  | mAb with HC and LC | 521 | **0.878** | 0.795 | 0.773 | 0.801 | 0.786 |
|  | monospecific mAb | 480 | **0.890** | 0.815 | 0.758 | 0.841 | 0.797 |
|  | bispecific mAb | 120 | 0.848 | 0.742 | 0.781 | 0.746 | 0.763 |
|  | all (val+test) | 600 | **0.883** | 0.798 | 0.760 | 0.818 | 0.788 |

^1^Combined analysis of September dataset's validation set and test set. "All (val+test)" refers to this aggregate, with other subsets examining mAbs by HC-only (VHH-Fc), HC+LC, monospecific (canonical IgG and monospecific VHH-Fc), or bispecific categories.

^2^Bold numbers indicate the best ROC AUC values in each subset.

Supp. Table 2. Model performance of PLM model 2 (Antibery-based) on 13 approved mAbs

See Excel File

Supp. Figure 1. Effect of mAb concentration on BVP and BSA ELISA. A total of 313 unique mAbs from July dataset were tested at both 667 nM and 66.7 nM. **A**: Percent of mAbs that showed polyreactivity (fold of signal over control greater than 2) in BVP and BSA ELISA at both concentrations. **B:** Percentage of mAbs that exhibited polyreactivity at 667 nM but appeared clean (fold of signal over control equal to or less than 2) at 66.7 nM.

Supp. Figure 2. Polyreactivity differences among allele families. **A** and **B**: Analysis of 228 unique monospecific mAbs (regular mAbs and VHH-Fc) tested at 667 nM in BVP and BSA ELISA. X-axis shows allele families. Y-axis in **A** shows fold over control, and in **B** shows number of mAbs per family. **C** and **D**: Analysis of 134 unique monospecific mAbs (regular mAbs only) tested at 667 nM in both assays. Axis labels are similar to **A** and **B**, except light chain families were shown instead.

Supp. Figure 3. Mechanistic insights. **A**: The five most important features in the descriptor model, as determined by the SHAP score. L1: LC CDR1. H1: HC CDR1. LFR3: LC FR3. Detailed information on the descriptors can be found on Schrodinger website. **B**: AlphaFold predicted structure (ribbons) and hydrophobic surfaces (green patches) for GP64 trimer. Views are shown looking sideways through the trimer surface (top) and down along the trimer surface (bottom).

Supp. Figure 4. Examples of sequence preprocessing. There are three steps in sequence preprocessing: stoichiometry-aware chain preprocess, variable region extraction, and variable region embedding **A**: Example of stoichiometry-aware chain preprocessing. **B**: Example of variable region extraction using the G4S linker approach. The extracted sequences were then embedded with a PLM model. **C**: Example of variable region extraction using the location matrix approach. The individual VH and VL regions were embedded with a PLM model. During the process, a location matrix was also generated to restore information about chain where the variable regions came from. This information was encoded as an integer between 0 and 4, denoting null, HC1, LC1, HC2, LC2 respectively.
